# Resting-State Neurophysiological Activity Patterns in Young People with ASD, ADHD, and ASD + ADHD

**DOI:** 10.1007/s10803-017-3300-4

**Published:** 2017-09-13

**Authors:** Elizabeth Shephard, Charlotte Tye, Karen L. Ashwood, Bahar Azadi, Philip Asherson, Patrick F. Bolton, Grainne McLoughlin

**Affiliations:** 1 MRC Social, Genetic and Developmental Psychiatry Centre, Institute of Psychiatry, Psychology, and Neuroscience, King’s College London, De Crespigny Park, London, SE5 8AF UK; 2 Department of Child and Adolescent Psychiatry, Institute of Psychiatry, Psychology, and Neuroscience, King’s College London, De Crespigny Park, London, SE5 8AF UK; 3 Present Address: Forensic and Neurodevelopmental Sciences, Institute of Psychiatry, Psychology, and Neuroscience, King’s College London, De Crespigny Park, London, UK

**Keywords:** ASD, ADHD, Co-occurring ASD + ADHD, Resting-state, EEG, Spectral power

## Abstract

Altered power of resting-state neurophysiological activity has been associated with autism spectrum disorder (ASD) and attention-deficit/hyperactivity disorder (ADHD), which commonly co-occur. We compared resting-state neurophysiological power in children with ASD, ADHD, co-occurring ASD + ADHD, and typically developing controls. Children with ASD (ASD/ASD + ADHD) showed reduced theta and alpha power compared to children without ASD (controls/ADHD). Children with ADHD (ADHD/ASD + ADHD) displayed decreased delta power compared to children without ADHD (ASD/controls). Children with ASD + ADHD largely presented as an additive co-occurrence with deficits of both disorders, although reduced theta compared to ADHD-only and reduced delta compared to controls suggested some unique markers. Identifying specific neurophysiological profiles in ASD and ADHD may assist in characterising more homogeneous subgroups to inform treatment approaches and aetiological investigations.

## Introduction

Autism spectrum disorder (ASD) and attention-deficit/hyperactivity disorder (ADHD) are two of the most common and impairing neurodevelopmental disorders which frequently co-occur and share genetic mechanisms (Grzadzinski et al. 2016; Ronald et al. 2008; Simonoff et al. 2008). It is not well understood whether the presence of both ASD and ADHD in one individual reflects a third distinct clinical entity, or if ASD and ADHD are different manifestations of a single entity. In order to understand the mechanisms underlying this overlap, it is important to characterise shared and/or distinct pathophysiological underpinnings. Both ASD and ADHD have been associated with atypicalities in brain structure and function (Ecker 2017; Friedman and Rapoport 2015). An ideal method of investigating the temporal dynamics of brain function in developmental psychiatric populations is through the measurement of electro-encephalographic (EEG) activity on the scalp. Resting-state EEG (activity recorded while the brain is not engaged in a specific task) is particularly well-suited to investigating brain function in developmental disorders due to the low cognitive demands required of the child. Neural indices obtained from resting-state EEG include the power of oscillations in different frequency bands, i.e. delta (0.5–3.5 Hz), theta (4–8 Hz), alpha (8–12 Hz), beta (12–30 Hz), and gamma (>30 Hz), which reveal information about baseline neurophysiological states such as motivation and neural excitability (Klimesch et al. 2007; Knyazev 2007).

Alterations in resting-state power of different frequency bands have been associated with ASD and ADHD. In turn, these alterations have been interpreted as reflecting neurophysiological disturbances core to the symptoms of ASD and ADHD. In ASD, both children and adults have been reported to show increased resting-state power in the slow delta and theta frequencies (Cantor et al. 1986; Chan et al. 2007; Cornew et al. 2012; Machado et al. 2015; Mathewson et al. 2012; Murias et al. 2007; Pop-Jordanova et al. 2010), reduced power in the middle-range alpha frequency (Cantor et al. 1986; Chan et al. 2007; Dawson et al. 1995; Machado et al. 2015; Murias et al. 2007), and increased power at high beta and gamma frequencies (Machado et al. 2015; Mathewson et al. 2012; Stroganova et al. 2007), compared to typically developing controls. This U-shaped EEG profile has been proposed to reflect an imbalance in cortical inhibition and excitability, such that reduced GABA-mediated cortical inhibition results in increased cortical excitation or hyper-arousal (Wang et al. 2013). In line with these interpretations, a cortical excitatory/inhibitory imbalance has been proposed to disrupt functional brain organisation in ASD, which in turn leads to the diverse social cognition, language, and emotional impairments characteristic of this disorder (Rubenstein and Merzenich 2003). Still, findings are inconsistent and may reflect clinical heterogeneity and developmental change.

In contrast, children and adults with ADHD have been reported to show increased resting-state activity in the slow frequencies, particularly the theta range (Barry et al. 2009; Bresnahan et al. 1999; Kitsune et al. 2015; Koehler et al. 2009; Tye et al. 2014), and less consistently, in the delta range (Bresnahan et al. 1999; Kitsune et al. 2015), compared to typically developing controls. The increased slow-wave activity is accompanied by decreased fast-wave activity in the beta range (Barry et al. 2009; Bresnahan et al. 1999; Buyck and Wiersema 2014; Clarke et al. 2006; Kitsune et al. 2015), and less reliably, in the alpha range (Barry et al. 2009; Clarke et al. 2006; Loo et al. 2009). These atypicalities have been interpreted as reflecting delayed brain maturation in ADHD (Barry et al. 2003), since delta/theta activity decreases with age in typical development (Klimesch 1999). Increased theta has also been suggested to reflect hypo-arousal and an under-focused, suboptimal energetic state (Sergeant 2000) or the action of a top-down attentional control network that regulates arousal level (Sergeant et al. 2003). In line with this interpretation, excessive resting-state theta and increased theta/beta ratio are associated with poor cognitive task performance in ADHD (Hermens et al. 2005; van Dongen-Boomsma et al. 2010). Nevertheless, the increased slow-wave + decreased fast-wave activity pattern may only present in a subset of individuals with ADHD (Arns 2012; Arns et al. 2008; Clarke et al. 2013) while other patterns of resting-state alterations, such as reduced alpha power, may characterise other individuals (Arns et al. 2008; Loo et al. 2009).

While the previous work in ASD and ADHD has contributed to understanding the neurobiological mechanisms involved in these disorders, one limitation is that few studies have controlled for comorbidity. A large proportion of individuals with ASD have co-occurring clinical or sub-clinical symptoms of ADHD and vice versa for individuals with ADHD (Grzadzinski et al. 2016; Simonoff et al. 2008; Tick et al. 2016). It is possible that some of the heterogeneity in resting-state EEG profiles in ASD and ADHD reflects unmeasured symptoms of the other disorder. An investigation of resting-state power in children with “pure” ASD (i.e. without co-occurring ADHD), “pure” ADHD (without co-occurring ASD), and children with co-occurring ASD and ADHD (ASD + ADHD) is needed to clarify the resting-state power atypicalities associated with ASD and ADHD, and to examine how atypicalities manifest in children with both disorders, that is, whether atypicalities are summed (“additive”) or whether there are more interactive effects of ASD and ADHD. An additive model suggests the single disorders (ASD-only, ADHD-only) can be differentiated from each other, but when the comorbid (ASD + ADHD) condition is considered the manifestations converge, such that the unique features are observed. Interactive models of ASD and ADHD may reflect the presence of independent subtypes, such that each disorder displays its own unique deficits with qualitatively distinct EEG profiles, or alternatively a symptomatic phenocopy, whereby ASD + ADHD presents with the same behavioural manifestation, but the EEG profile is similar to ASD and not ADHD (or vice versa; Banaschewski and Brandeis 2007). Accordingly, a direct comparison across the three patient groups enables a test of the model of comorbidity, which can provide insight into the brain-behaviour pathways in each disorder and inform previous inconsistent associations between EEG profiles and behaviour. There are limited comparisons of resting-state neurophysiological activity in children with ASD + ADHD. Previous inconsistent studies have reported elevated beta power in children with ADHD and co-occurring ASD traits compared to children with ADHD-only (Clarke et al. 2011), or increased resting-state theta power in adolescents with ADHD-only compared to adolescents with ASD + ADHD, interpreted as reflecting hypoarousal in the adolescents with ADHD without ASD compared to those with ASD + ADHD (Bink et al. 2015). However, these study designs do not enable a test of whether differences in the comorbid group reflect co-occurring ASD symptoms or rather the effects of multiple neurodevelopmental pathophysiologies.

In the current study we aimed to address the limitations with the previous work by examining resting-state neurophysiological activity in children with pure ASD, pure ADHD, ASD + ADHD, and typically developing children. We aimed to clarify the profile of resting-state atypicalities in ASD, ADHD, and ASD + ADHD. Further, we sought to assess how ASD- and ADHD-related resting-state atypicalities manifest in children with both disorders, that is, whether the atypicalities are additive or interactive in children with ASD + ADHD. We hypothesised that, firstly, children with ASD would show a U-shaped pattern of resting-state neurophysiological abnormality, with increased delta, theta, and beta power but decreased alpha power compared to controls. Secondly, children with ADHD would show increased slow-wave (delta and theta) activity and decreased fast-wave (alpha and beta) activity compared to controls. Finally, we predicted that children with ASD + ADHD would show additive ASD- and ADHD-related atypicalities in resting-state power, characterised by increased delta and theta power and reduced alpha power compared to controls, as well as decreased beta power compared to the ASD group but increased beta power compared to the ADHD group. This would suggest that the co-occurring symptoms reflect true overlap between ASD and ADHD at the neurophysiological level.

## Methods

### Participants

Participants were boys aged 8–13 years with ASD (ASD group: *n* = 19), ADHD (ADHD group: *n* = 18), or ASD + ADHD (ASD + ADHD group: *n* = 29), and typically developing boys (Control group: *n* = 26). We included only male participants to reduce sample heterogeneity. Group characteristics are presented in Table 1. All participants had normal or corrected-to-normal vision, IQ scores in the normal range (>69 on the Wechsler Abbreviated Scale of Intelligence; Wechsler 1999), and were without neurological conditions or co-occurring neurodevelopmental/psychiatric conditions other than ASD/ADHD (excluding oppositional defiant disorder). Participants were excluded from the study if they were receiving medications other than stimulants. Six boys with ADHD and six boys with ASD + ADHD were receiving stimulant medication; all 12 children refrained from taking their medication for 48 h prior to testing. Boys with ASD and/or ADHD were recruited from South London neurodevelopmental outpatient clinics and held a DSM-IV (American Psychiatric Association 2000) or ICD-10 (World Health Organisation 1993) clinical diagnosis of one or both disorders. Research diagnoses were confirmed by trained researchers using the social communication questionnaire (SCQ) (Rutter et al. 2003), autism diagnostic interview-revised (ADI-R) (Lord et al. 1994) and autism diagnostic observation schedule-generic (ADOS-G) (Lord et al. 2000) for ASD, and the conners 3 parent short form (Conners 2008) and parental account of childhood symptoms (PACS) (Taylor et al. 1986) for ADHD. Typically developing boys without neurodevelopmental or psychiatric diagnoses and without siblings with ASD or ADHD were recruited from local schools and forums for the control group. All control participants were screened for subclinical symptoms using the strengths and difficulties questionnaire (SDQ) (Goodman 1997), SCQ, and Conners 3. Ethical approval for the study was obtained from the NHS National Research Ethics Service (NHS RES Wandsworth REC 08/H0903/161) and London Research and Development Departments. In accordance with the declaration of Helsinki, parental written informed consent was obtained prior to completion of study measures.

**Table 1:** Group characteristics

	ASD (*n* = 19)	ADHD (*n* = 18)	ASD + ADHD (*n* = 29)	Controls (*n* = 26)	Group differences
Age (months)	138.42 (19.44)^a^	116.56 (21.60)^b^	120.62 (20.40)^b^	124.12 (22.84)	*F*(3, 88) = 3.91, *p* = .01, *η* ^2^ = 0.118
WASI FSIQ	115.42 (15.52)	104.11 (13.84)^a^	109.41 (13.29)^a^	120.04 (13.42)^b^	*F*(3, 88) = 5.48, *p* = .002, *η* ^2^ = 0.157
Hyp/Imp	66.11 (12.99)^a^	87.89 (3.25)^b^	84.24 (7.71)^b^	58.73 (17.12)^a^	*F*(3, 88) = 33.07, *p* < .001, *η* ^2^ = 0.530
Inattention	67.11 (14.13)^a^	83.94 (7.41)^b^	81.10 (9.85)^b^	56.27 (10.89)^c^	*F*(3, 88) = 34.06, *p* < .001, *η* ^2^ = 0.537
SCQ	20.37 (6.80)^a^	10.89 (5.36)^b^	24.59 (5.39)^a^	3.73 (3.62)^c^	*F*(3, 88) = 81.27, *p* < .001, *η* ^2^ = 0.735

Groups marked with different superscript letters (a–c) differed significantly with Bonferroni correction applied (*p* < .05)

*WASI FSIQ* Wechsler Abbreviated Scale of Intelligence-Full-Scale IQ, *Hyp*/*Imp* and *Inattention* conners 3 parent-rated short form hyperactivity/impulsivity and inattentive T-scores, *SCQ* social communication questionnaire total score

### Eyes-Open Resting-State Paradigm

Participants completed 6 min of eyes-open (EO) resting-state EEG during a 1-h EEG task battery, which also included 6 min of eyes-closed resting state and a series of experimental tasks (data not presented here). During the EO resting-state, participants fixated on a dot on the opposite wall and were encouraged to minimise ocular and other movements.

### EEG Acquisition and Processing

EEG was recorded continuously from 62 Ag/AgCl active (actiCAP) scalp electrodes placed according to the extended 10–20 system using an ActiCHamp (active channel amplifier) DC-coupled Brain Products recording system (Brain Products, Munich, Germany). The data were referenced online to electrode FCz and sampled at 500 Hz. Electrode impedances were kept below 10 kΩ. Vertical and horizontal eye movements were recorded from electrodes placed above and below the left eye and at the outer canthi. EEG data were processed offline using Brain Vision Analyzer v2.03 (Brain Products, Munich, Germany). Flat or noisy channels were removed and interpolated using spherical spline interpolation prior to re-referencing to the average reference and filtering with 0.1 Hz high-pass, 30 Hz low-pass, 50 Hz notch Butterworth 24dB/Oct filters. Independent components analysis (ICA) was used to identify and remove ocular artefact components after which the data were segmented into 2-second non-overlapping epochs within the EO condition. Automated artefact-detection was used to exclude any epochs with remaining artefacts, defined as those with amplitudes exceeding ±90 µv or a peak-to-peak amplitude change of 200 µv; this resulted in the exclusion of between 1 and 166 epochs (2–332 s/0.6–92% of the EO data) across participants (mean number of epochs removed in the sample = 51.36, SD = 44.92). Clean epochs were subjected to Fast Fourier Transform (FFT) with a 10% Hanning window taper to obtain absolute spectral power in the delta (0.5–3.5 Hz), theta (4–8 Hz), alpha (8–12 Hz) and beta (12–20 Hz) frequency bands. Following previous research (Liechti et al. 2013; Loo and Smalley 2008), absolute power in each band/condition was averaged over clusters of electrodes at frontal (F1–F8, Fz), central (C1–C6, Cz), parietal (P3–4, P7–8, Pz), and occipital (O1–2, Oz) scalp locations for analysis. Power data were log-transformed to approximate a normal distribution prior to statistical analysis.

Participants were excluded from analyses if they had <40 s of artefact-free data (20 epochs) according to the artefact-rejection criteria described above, or had power values (prior to log transform) 3.5SD outside of their group mean. Three control boys, one boy with ASD, three boys with ADHD, and four boys with ASD + ADHD were excluded due to having insufficient artefact-free epochs for analysis (<15 epochs). The EEG data from these children was characterised by excessive muscular artefact and slow waves. A further one control boy, two boys with ASD, and four boys with ASD + ADHD were excluded for having outlying power values in multiple frequency bands. The analysis of resting-state power was therefore conducted on a final sample of 22 Controls, 16 ASD, 15 ADHD, and 21 ASD + ADHD. The number of epochs included in analysis did not differ between groups [*F*(3, 70) = 0.68, *p* = .58, *ηp* ^2^ = 0.027; ASD mean (SD) = 123.94 (45.88), ADHD mean (SD) = 126.80 (47.57), ASD + ADHD mean (SD) = 140.76 (42.78), Control mean (SD) = 139.95 (45.88)]. The majority of the children included in the final analysis had at least 45 epochs (90 s) of artefact-free data for analysis; one child with ASD + ADHD had only 23 epochs. Statistical analyses were repeated without this child and results are reported wherever they differ from the main analyses.

### Statistical Analysis

Statistical analyses were conducted in SPSS v22 (IBM Corp 2013). We tested the hypothesised atypicalities in resting-state power in ASD, ADHD, and ASD + ADHD in two ways. Firstly, to assess the profile of resting-state EEG power in each participant group, we used ANCOVA to compare power in each frequency band between the four groups (ASD, ADHD, ASD + ADHD, Controls). A separate model was used for each frequency band (delta, theta, alpha, beta). All models included electrode cluster (frontal, central, parietal, occipital) as a within-subjects factor. Significant main effects of group and cluster, and interactions between these factors, were further investigated using planned pairwise contrasts between pairs of groups/clusters with Bonferroni correction applied to control for multiple comparisons. Secondly, we used a factorial approach to allow us to test for effects of ADHD (both ADHD groups compared to both non-ADHD groups) and ASD (both ASD groups compared to both non-ASD groups) and the interaction between these factors on resting-state power. The test for the interaction between ASD and ADHD factors was crucial for testing the hypothesis that ASD + ADHD reflects additive comorbidity. For this analysis, power in each frequency band was entered into 2 × 2 factorial ANCOVAs with the between-subjects factors ASD (ASD-yes: ASD and ASD + ADHD groups; ASD-no: ADHD and Control groups) and ADHD (ADHD-yes: ADHD and ASD + ADHD groups; ADHD-no: ASD and Control groups). Electrode cluster (frontal, central, parietal, occipital) was entered as a within subjects factor in all models. A separate model was used for power in each of the four frequency bands. Significant main effects of ASD, ADHD, and cluster, and significant interactions between these factors, were further investigated using planned pairwise contrasts with Bonferroni correction applied to control for multiple comparisons. IQ and age were included as covariates in all models given known effects of these variables on EEG power (Kitsune et al. 2015; Michels et al. 2013).

Finally, we conducted a dimensional analysis to investigate how symptoms of ASD and ADHD were associated with resting-state power in the whole sample. Pearson correlation coefficients were computed between SCQ scores (ASD symptoms), Conners Hyperactive/Impulsive and Inattentive T-scores (ADHD symptoms) and resting-state power values. Only power in frequency bands that differed significantly between groups were included in dimensional analysis to limit the number of tests conducted.

## Results

**Fig. 1:**
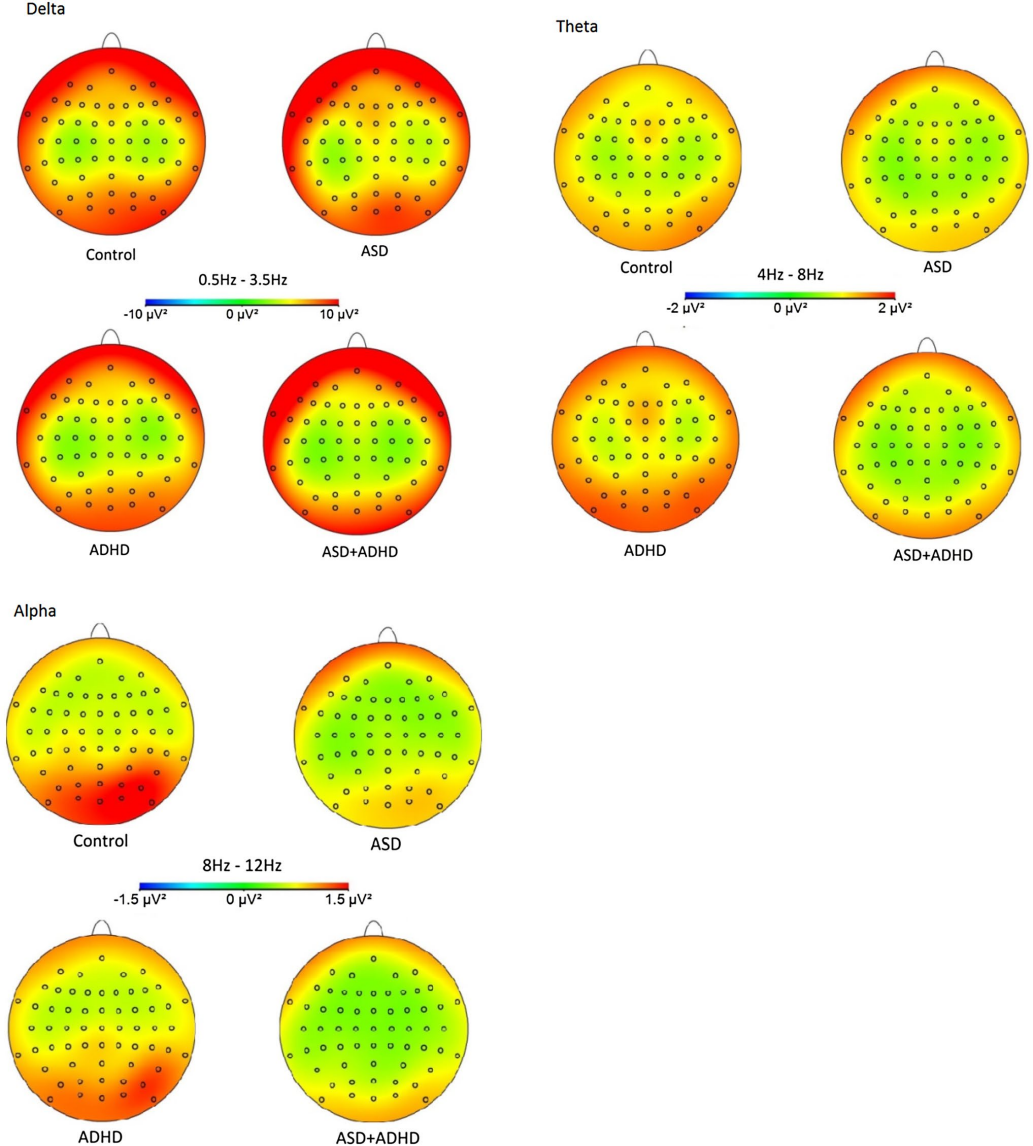
Grand-averaged absolute power (prior to log transform) in the delta (0.5–3.5 Hz). theta (4–8 Hz), and alpha (8–12 Hz) bands for each participant group

**Fig. 2:**
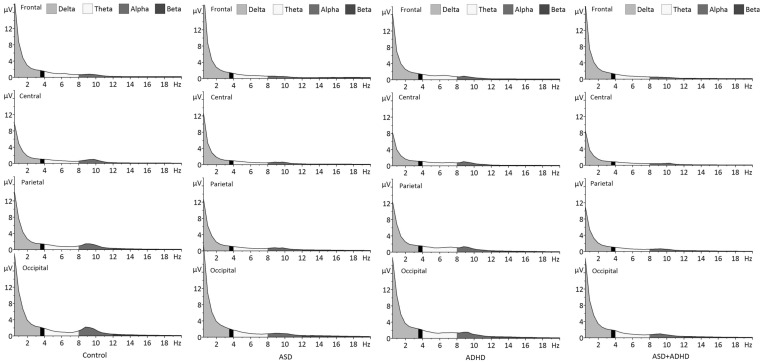
Grand-averaged spectral power (prior to log transform) at each electrode cluster (frontal, central, parietal, occipital) in the delta (0.5–3.5 Hz), theta (4–8 Hz), alpha (8–12 Hz), and beta (12–20 Hz) bands for each participant group

Absolute power values (prior to log transform) are presented by group in Table 2. Grand averaged absolute power values (prior to log transform) are presented by group in Figs. 1 and 2.

**Table 2:** Mean (SD) absolute power values (µ^2^) by group

	ASD (*n* = 16)	ADHD (*n* = 15)	ASD + ADHD (*n* = 21)	Controls (*n* = 22)
Delta				
Frontal	7.14 (1.87)	5.58 (1.78)	6.09 (1.80)	6.59 (2.16)
Central	4.28 (1.36)	3.13 (1.50)	3.04 (0.78)	3.70 (1.07)
Parietal	4.81 (1.89)	4.83 (2.04)	4.11 (0.98)	5.49 (1.78)
Occipital	8.49 (4.37)	7.47 (2.98)	7.13 (1.72)	7.82 (3.06)
Theta				
Frontal	0.85 (0.25)	1.07 (0.51)	0.84 (0.33)	1.01 (0.39)
Central	0.65 (0.22)	0.85 (0.54)	0.58 (0.20)	0.75 (0.25)
Parietal	0.75 (0.32)	1.19 (0.80)	0.73 (0.23)	0.97 (0.37)
Occipital	1.11 (0.46)	1.51 (0.80)	1.08 (0.37)	1.18 (0.40)
Alpha				
Frontal	0.49 (0.20)	0.63 (0.35)	0.44 (0.15)	0.60 (0.26)
Central	0.45 (0.29)	0.67 (0.53)	0.39 (0.21)	0.68 (0.39)
Parietal	0.61 (0.33)	0.98 (0.67)	0.53 (0.21)	0.99 (0.69)
Occipital	0.85 (0.37)	1.25 (0.95)	0.74 (0.29)	1.39 (1.02)
Beta				
Frontal	0.20 (0.07)	0.19 (0.11)	0.18 (0.06)	0.19 (0.08)
Central	0.13 (0.07)	0.14 (0.10)	0.10 (0.04)	0.11 (0.06)
Parietal	0.18 (0.11)	0.20 (0.11)	0.15 (0.05)	0.17 (0.10)
Occipital	0.28 (0.13)	0.28 (0.11)	0.21 (0.07)	0.23 (0.11)

Absolute power values (µ^2^) in the EO resting-state are presented by group, prior to log-transform. Delta: 0.5–3.5 Hz, theta: 4–8 Hz, alpha: 8–12 Hz, beta: 12–20 Hz

### Delta Range

There was a significant main effect of electrode cluster [*F*(2.46, 167.52) = 3.16, *p* = .04, *ηp* ^2^ = 0.044] and a significant group*cluster interaction [*F*(7.39, 167.52) = 4.03, *p* < .001, *ηp* ^2^ = 0.151] on absolute delta power. The main effect of cluster reflected significant differences in delta power between all pairs of electrode clusters (all *p* < .001), with power greatest at occipital and frontal scalp. The group*cluster interaction reflected significantly lower delta power in the ADHD group than the ASD group at the frontal cluster (*p* = .03, *d* = 0.85), significantly lower power in both ADHD and ASD + ADHD groups than the ASD group at the central cluster (both *p* < .001, *d* ≥ 0.80), and significantly lower power in the ASD + ADHD group than the Control group at the parietal cluster (*p* = .03, *d* = 0.96) (Figs. 1, 2). When combined by ASD/ADHD diagnosis, there was a significant main effect of ADHD [*F*(1, 68) = 6.40, *p* = .01, *ηp* ^2^ = 0.086], reflecting significantly lower delta power in boys with ADHD than those without ADHD. This effect was qualified by a significant ADHD*cluster interaction [*F*(2.46, 167.52) = 7.31, *p* < .001, *ηp* ^2^ = 0.097], which showed that the reduction in delta power in boys with ADHD was significant at frontal (*p* = .008, *d* = 0.49), central (*p* < .001, *d* = 0.75), and parietal (*p* = .03, *d* = 0.47) clusters. There was also a significant ASD*cluster interaction [*F*(2.46, 167.52) = 4.74, *p* = .006, *ηp* ^2^ = 0.065], which revealed a trend for increased delta power in boys with ASD compared to boys without ASD at the frontal cluster (*p* = .07, *d* = 0.18). The interaction between the ASD and ADHD factors was non-significant [*F*(1, 68) = 1.04, *p* = .31, *ηp* ^2^ = 0.015] supporting additive effects of ASD and ADHD. Age [*F*(1, 68) = 9.09, *p* = .004, *ηp* ^2^ = .118] but not IQ (*p* = .11, *ηp* ^2^ = .036) was a significant covariate in these models.

### Theta Range

There was a significant main effect of cluster on absolute theta power [*F*(2.49, 168.98) = 4.40, *p* = .009, *ηp* ^2^ = 0.061], reflecting significant differences between all pairs of electrodes (all *p* ≤ .05) with power largest at frontal and occipital scalp, and a marginal main effect of group [*F*(3, 68) = 2.71, *p* = .052, *ηp* ^2^ = 0.107], reflecting a trend for greater theta power in the ADHD than ASD + ADHD group (*p* = .06, *d* = 0.71) (Figs. 1, 2). These main effects were qualified by a significant group*cluster interaction [*F*(7.46, 168.98) = 3.01, *p* = .004, *ηp* ^2^ = 0.117], which showed that the ADHD group had significantly greater theta power than the ASD + ADHD group at parietal scalp (*p* = .03, *d* = 0.78). Combining the groups by ASD/ADHD diagnosis, there was a significant main effect of ASD [*F*(1, 68) = 4.81, *p* = .03, *ηp* ^2^ = 0.066], which reflected significantly lower theta power in boys with ASD than boys without ASD. There was also a significant ADHD*cluster interaction [*F*(2.49, 168.98) = 6.20, *p* = .001, *ηp* ^2^ = 0.084], but follow-up Bonferroni-corrected pairwise group contrasts at each electrode cluster separately revealed no significant differences between boys with ADHD and those without ADHD (all *p* ≥ .16). The ASD*ADHD interaction was non-significant [*F*(1, 68) = 2.45, *p* = .12, *ηp* ^2^ = 0.035], supporting additive effects. Age was a significant covariate in these models [*F*(1, 68) = 17.86, *p* < .001, *ηp* ^2^ = 0.208], while IQ was non-significant (*p* = .19, *ηp* ^2^ = 0.025).

### Alpha Range

There was a significant main effect of group on absolute alpha power (*F*(3, 68) = 3.84, *p* = .01, *ηp* ^*2*^ = 0.145), reflecting a trend for lower power in the ASD + ADHD group than in the ADHD group (*p* = .06, *d* = 0.86) (Figs. 1, 2). Combined by ASD/ADHD diagnosis, there was a significant main effect of ASD (*F*(1, 68) = 10.91, *p* = .002, *ηp* ^*2*^ = 0.138), reflecting significantly lower alpha power in boys with ASD than boys without ASD. The ASD*ADHD interaction was non-significant (*F*(1, 68) = 0.218, *p* = .64, *ηp* ^*2*^ = 0.003), suggestive of additive effects. Age and IQ were non-significant covariates in these models (both *p* ≥ .48, *ηp* ^*2*^ ≤ 0.008).

### Beta Range

There was a significant main effect of cluster [*F*(2.29, 155.52) = 3.01, *p* = .045, *ηp* ^2^ = 0.042] on absolute beta power, reflecting greater power at the central cluster than all other clusters (all *p* ≤ .001), as well as a significant group*cluster interaction [*F*(6.86, 155.52) = 2.26, *p* = .03, *ηp* ^2^ = 0.090]. However, further investigation of this interaction revealed no significant differences between the four groups at any of the clusters (all *p* ≥ .46). There were no significant main effects of ASD or ADHD and no interaction between these factors when combined by diagnosis (all *F* ≤ 2.86, *p* ≥ .10, *ηp* ^2^ ≤ 0.040). Age and IQ were non-significant covariates (both *F* ≤ 0.896, *p* ≥ .35, *ηp* ^2^ ≤ 0.013).

### Associations Between Resting-State Power and Symptoms of ASD and ADHD

SCQ scores were significantly negatively correlated with delta power at the parietal cluster [*r*(74) = −0.334, *p* = .004, *r* ^2^ = 0.11], theta power at frontal [*r*(74) = −0.239, *p* = .04, *r* ^2^ = 0.06], central [*r*(74) = −0.301, *p* = .009, *r* ^2^ = 0.09], and parietal [*r*(74) = −0.332, *p* = .004, *r* ^2^ = 0.11] clusters, and with alpha power at frontal [*r*(74) = −0.381, *p* = .001, *r* ^2^ = 0.15], central [*r*(74) = −0.468, *p* < .001, *r* ^2^ = 0.22], parietal [*r*(74) = −0.440, *p* < .001, *r* ^2^ = 0.19], and occipital [*r*(74) = −0.387, *p* = .001, *r* ^2^ = 0.15] clusters, indicating children with higher levels of ASD symptoms or traits had lower delta, theta, and alpha power. There were no significant associations between hyperactive/impulsive or inattentive symptoms of ADHD and power at any frequency or cluster (all *r* ≤ −.209, all *p* ≥ .07, all *r* ^2^ ≤ 0.04).

## Discussion

This study examined neurophysiological activity during the resting-state in children with ASD, ADHD, and co-occurring ASD + ADHD compared to typically developing controls. The findings appear to dissociate ASD and ADHD on the basis of different neurophysiological power profiles. Specifically in relation to our hypotheses, (1) children with ASD showed reduced theta and alpha power compared to children without ASD; (2) children with ADHD showed decreased delta power compared to children without ADHD; and (3), children with ASD + ADHD displayed a largely additive profile with the unique deficits of both ASD and ADHD, although specific differences compared to “pure” cases of ASD and ADHD were also observed.

Children with ASD (ASD/ASD + ADHD) demonstrated a unique EEG profile of reduced power in the theta and alpha frequencies compared to children without ASD. This pattern partially contrasts with previous suggestions of a U-shaped profile in ASD with increased power at low (delta, theta) and high (beta) frequencies and reduced alpha power (Wang et al. 2013). Nevertheless, our finding of reduced alpha power is in line with several previous resting-state studies of children (Cantor et al. 1986; Chan et al. 2007; Cornew et al. 2012) and adults with ASD (Mathewson et al. 2012; Murias et al. 2007), suggesting this atypicality may be a robust characteristic of individuals with ASD. Further, alpha power at all electrode clusters was negatively associated with SCQ scores, indicating that children with more severe ASD symptoms or traits had greater reductions in alpha power. We interpret these findings as being in line with the excitatory/inhibitory imbalance hypothesis of ASD (Rubenstein and Merzenich 2003) since alpha de-synchronisation (decreased alpha power) is associated with decreased tonic neural inhibition/increased neural excitability (Klimesch et al. 2007) and the idling state of alpha oscillatory activity has been directly linked with GABAergic circuitry (Jensen and Mazaheri 2010), which modulates excitatory cell activity. Further, inhibitory interneurons, which are likely abnormal in ASD (Casanova et al. 2002), play a role in maintaining alpha oscillations (Lőrincz et al. 2009). Longitudinal studies will be necessary to investigate whether the excitatory/inhibitory imbalance occurs early in development and if this pattern reflects a core pathophysiology in ASD. It will also be important for future work to investigate relationships between resting-state alpha power and social cognition, emotion processing, and language ability in ASD to fully test the proposed causal links between alpha oscillations, excitatory/inhibitory imbalance, functional brain disruption, and cognition (Rubenstein and Merzenich 2003; Thatcher et al. 2009; Wang et al. 2013). Nevertheless, the robust reductions in alpha power in the current and previous studies, as well as the strong negative association between ASD symptoms and alpha reductions, suggest that this neurophysiological atypicality may be a useful target for treating ASD symptoms, for example via neurofeedback training or as a biomarker in clinical drug trials.

Reduced theta power in children with ASD is consistent with some previous research on children (Dawson et al. 1995; Machado et al. 2015), although the majority of previous studies have found increased theta power in children and adults with ASD (Coben et al. 2008; Cornew et al. 2012; Mathewson et al. 2012; Murias et al. 2007), indicating alterations in resting-state theta activity are more heterogeneous in ASD than are atypical alpha oscillations. Since we observed the reduced theta in children with ASD with and without co-occurring ADHD symptoms, it is unlikely that the inconsistency in theta alterations across studies reflects the influence of unmeasured comorbidity with ADHD. Theta at frontal, central, and parietal scalp was negatively correlated with SCQ scores, indicating that, in our sample, reduced theta was associated with increased ASD symptoms dimensionally as well as at the group level. Decreases in theta power have been associated with increases in arousal levels in typically developing children (Barry et al. 2009). One interpretation of our theta finding is therefore that it reflects hyper-arousal. This interpretation is consistent with previous findings of hyper-arousal in ASD as indexed by skin conductance and pupillometry measures, and with models that propose some of the symptoms of ASD, particularly sensory abnormalities, reflect attempts to control over-arousal (Hirstein et al. 2001; Martineau et al. 2011; Prince et al. 2016). Since not all children with ASD exhibit significant sensory abnormalities, this might explain the heterogeneity in theta alterations across studies. It will be important for future research to test this hyper-arousal interpretation further by examining associations between theta power, skin conductance or pupillometry measures of arousal, and sensory symptoms in ASD.

Children with ADHD (ADHD/ASD + ADHD) showed reduced delta power in fronto-central and parietal regions compared to children without ADHD. Previous work has found either no differences in delta between children and adults with ADHD and controls (Buyck and Wiersema 2014; Clarke et al. 2006; Koehler et al. 2009), or increased delta in adolescents and adults with ADHD compared to controls (Bresnahan et al. 1999; Kitsune et al. 2015). Our four-group analysis indicated reduced delta power in the ADHD groups compared to the ASD-only group across fronto-central scalp regions (in line with the full-factorial results), thus group effects may reflect differences between clinical groups rather than case-control differences. In support, a trend towards elevated delta power in children with ASD diagnosis at frontal scalp regions was indicated, compared to children without ASD diagnosis, in addition to effects of ADHD diagnosis. Delta oscillations have been associated with function of the default mode network (DMN), the idling network of the brain which is prominent during rest and becomes deactivated during cognitive tasks. For instance, using simultaneous EEG-fMRI, Hlinka, Alexakis, Diukova, Liddle, and Auer (2010) reported a negative association between delta power and connectivity within the DMN in typical adults. In line with this, our reduced delta finding could reflect alterations in functional connectivity within the DMN in children with ADHD. This interpretation is consistent with MRI research indicating that DMN connectivity is altered in ADHD (Uddin et al. 2008) and models proposing that atypical DMN connectivity is involved in causing attentional problems in ADHD (Sonuga-Barke and Castellanos 2007). However, we did not find that delta power was associated with inattentive symptoms in dimensional analysis. Delta activity has also been proposed to index baseline activity in dopaminergic reward/reinforcement circuitry (Knyazev 2007). Reduced delta power in ADHD may therefore reflect tonic hypo-activity in this circuitry, which is in line with models proposing that hypo-dopaminergia and impaired reinforcement/reward processing are core to the pathology of ADHD (Sagvolden et al. 2005) as well as empirical findings of impaired behavioural performance and atypical neurophysiological correlates of reinforcement learning in ADHD (Frank et al. 2007; Shephard et al. 2016). Further research is needed to clarify the role of atypical resting-state delta oscillations in ADHD, for example by correlating delta power during rest with DMN connectivity assessed with fMRI and with reinforcement learning task performance.

In contrast to our hypotheses and many previous studies (Barry et al. 2009; Bresnahan et al. 1999; Kitsune et al. 2015; Koehler et al. 2009; Tye et al. 2014), we found limited evidence for increased theta activity in children with ADHD compared to typically developing children. However, several recent studies of both children and adults with ADHD have failed to replicate past findings of increased theta in ADHD (Buyck and Wiersema 2014; Liechti et al. 2013; Loo et al. 2009; van Dongen-Boomsma et al. 2010), which together with the current findings questions the legitimacy of increased resting-state theta as a marker of ADHD. A recent study demonstrated that theta power is comparable between adults with ADHD and controls during the resting-state, but changes (increases) in theta power from the resting-state to cognitive task-states is diminished in ADHD compared to controls, and this pattern normalises with methylphenidate treatment and associated improvements in ADHD symptoms (Skirrow et al. 2015). Thus, it may be the case that alterations in task-related theta activity, rather than baseline theta oscillations, are associated with the ADHD phenotype (McLoughlin et al. 2014).

Our findings converge to suggest a dissociation between ASD and ADHD on the basis of their cortical EEG profiles, whereby children with ASD display a high delta, low theta and low alpha pattern and children with ADHD display a low delta pattern. Importantly, children with ASD + ADHD largely present as an additive co-occurrence of both ASD and ADHD, with low delta, low theta and low alpha patterns, rather than presenting as a distinct entity with unique patterns of EEG power. The finding that the ASD + ADHD group presents with the unique deficits of both disorders suggests it cannot be assumed that the correlates and aetiology of ASD are the same regardless of the presence of absence or ADHD, and vice versa (Caron and Rutter 1991). This has implications both for assessing and treating individuals with both conditions (as treatment of ADHD may not reduce ASD symptoms), and in the identification of more homogenous subgroups to further understand genetic and biological underpinnings and to target specific treatments. However, the children with ASD + ADHD also displayed reduced theta power compared to children with pure ADHD at parietal scalp regions (in line with Bink et al. 2015) and reduced delta power compared to typically developing children in parietal regions, which indicates some unique patterns of EEG power compared to the single disorders. The reduction of power across all frequency bands in ASD + ADHD may suggest qualitative differences in resting brain activity across pure and comorbid cases. Still, there was no evidence of non-additive statistical interactions between ASD and ADHD diagnosis to support the comorbid condition as a qualitatively distinct entity, although this may reflect limited power to detect significant interactions. This suggests that resting-state EEG profiles in ASD and in ADHD are not dependent on or exacerbated by having the comorbidity, but rather EEG profiles in the comorbid group are the product of both conditions.

Several limitations should be taken into consideration. The small sample size limits firm conclusions and along with heterogeneity in EEG profiles may have contributed to null findings. For example, lack of group differences on beta power may reflect the presence of distinct EEG subtypes that have been described in children with ADHD, that differ on deficiency versus excess beta power (Clarke et al. 2001) and behavioural subtypes within children with ASD that differ on alpha power (Dawson et al. 1995). It will be important to investigate changes in power and group differences under different conditions, including real-world contexts. For example, part of the EEG here was collected at the beginning of the testing session and therefore may reflect the potential anxious state of the child (e.g. reduced alpha) in a new clinical environment. In support, EEG findings are different when recorded at the beginning compared to the end of a testing session in adolescents and adults with ADHD (Kitsune et al. 2015). The topographical differences observed in the delta and theta bands warrant further research using advanced source analysis of EEG data (McLoughlin et al. 2014). This may, for example, reflect group differences in connectivity between brain regions that are not captured by absolute power indices at selected scalp regions. An additional consideration is differing developmental trajectories in relation to EEG power (and potential compensatory processes in the examination of associated changes in EEG power). For example, a recent review indicates developmental subtypes of ASD and ADHD may be related to changing connectivity in frontal brain regions with age (Rommelse et al. 2017). Future longitudinal studies that track oscillatory power at frequent intervals will help to characterise the developmental changes to disorder specificity in EEG profiles, across a range of disorders associated with altered EEG profiles. A final consideration is that the power values we report (see Table 2) are smaller than those reported in some studies (e.g. Barry et al. 2009; Liechti et al. 2013; Loo et al. 2009), although other previous studies have reported similarly low power values (e.g. Kitsune et al. 2015; Tye et al. 2014; van Dongen-Boomsma et al. 2010). We could not identify a systematic difference in EEG recording or processing parameters that could explain the variation in power values across studies, and we do not have reason to believe that our lower power values contributed to group differences in power, but it might be helpful for future research to systematically explore the effects of different amplifier and processing settings on resting-state power to resolve this inconsistency in the field. This would be particularly helpful for multi-centre studies comparing EEG data collected with different recording systems across participant groups.

In conclusion, this study extends previous studies of EEG power in ASD and ADHD by identifying distinct profiles, while demonstrating that children with comorbid ASD + ADHD largely demonstrate the unique deficits of both disorders. Examination of EEG power at rest is therefore useful in elucidating the basis of these overlapping neurodevelopmental disorders and the potential biological pathways that underlie comorbidity. Such findings are likely to show clinical value by aiding in evaluating the validity of non-invasive EEG in the diagnosis and targeted treatment of more homogenous subgroups of neurodevelopmental disorders, and informing aetiological investigations.
